# An in vitro assay of the effect of lysine oxidation end-product, α-aminoadipic acid, on the redox status and gene expression in probiotic *Lactobacillus reuteri* PL503

**DOI:** 10.1007/s00726-021-03087-4

**Published:** 2021-10-17

**Authors:** Patricia Padilla, María J. Andrade, Fernando J. Peña, Alicia Rodríguez, Mario Estévez

**Affiliations:** 1 Food Technology, IPROCAR Research Institute, University of Extremadura, 10003 Cáceres, Spain; 2 Faculty of Veterinary Science, IPROCAR Research Institute, Food Hygiene and Safety, University of Extremadura, 10003 Cáceres, Spain; 3 Laboratory of Equine Reproduction and Equine Spermatology, University of Extremadura, 10003 Cáceres, Spain

**Keywords:** Oxidized amino acids, Oxidative stress, Probiotic bacterium, Protein oxidation, Transcripts

## Abstract

This study was designed to gain information about the underlying mechanisms of the effects of a food-occurring free oxidized amino acid, α-aminoadipic acid (AAA), on the probiotic *Lactobacillus reuteri* PL503. This bacterium was incubated in colonic-simulated conditions (37 °C for 24 h in microaerophilic conditions) and exposed to three food-compatible AAA concentrations, namely, 1 mM, 5 mM, and 10 mM. A control group with no AAA exposure was also considered. Each of the four experimental conditions was replicated three times and samplings were collected at 12, 16, 20, and 24 h. The downregulation of the *uspA* gene by AAA (0.5-fold decrease as compared to control) suggests that AAA is identified as a potential chemical threat. The *dhaT* gene, implicated in the antioxidant defense, was found to be upregulated in bacteria treated with 1 and 5 mM AAA (up to twofold increase, as compared to control), which suggest the ability of the oxidized amino acid to impair the redox status of the bacterium. In fact, AAA caused an increased production of reactive oxygen species (ROS) and the accretion of post-translational changes (protein carbonylation) in *L. reuteri* (up to 13 nmol allysine/mg protein vs 1.8 nmol allysine/mg protein in control). These results suggest that probiotic bacteria identify oxidized amino acids as harmful species and activate mechanisms that may protect themselves and the host against their noxious effects.

## Introduction

Protein oxidation is a post-translational modification induced by reactive oxygen species (ROS) and other pro-oxidative compounds, and plays an essential role in the pathogenesis of relevant degenerative diseases (Davies 2005). The oxidative damage to proteins leads to depletion of original amino acids and the formation, in its place of specific oxidation products (Davies 2005). Chemical species such as the α-aminoadipic semialdehyde (AAS), also known as allysine, and its end-product, the α-aminoadipic acid (AAA), are generated by the oxidation of lysine through metal-catalyzed reactions (Stadtman and Oliver 1991). While both species occur as intermediates in lysine metabolism, the accretion of such species in biological samples, including food systems, respond to a radical-mediated oxidation mechanism (Davies 2005; Stadtman and Oliver 1991). The chemical structures and formation mechanisms of these oxidized amino acids can be found in detail elsewhere (Luna et al. 2021). AAA has been identified in meat products, such as raw and cooked patties, cooked sausages and fermented meats, at levels ranging 50–200 µM (Utrera and Estévez 2012; Utrera et al. 2012). Recently, Estévez and Xiong (2019) collected information about the scientific evidences of the potential harmful effects of dietary oxidized proteins and amino acids. AAA, in particular, has been found to exert, at food-compatible concentrations of AAA (200 µM), mitochondrial disturbance, oxidative stress, apoptosis, and necrosis in human intestinal and mice pancreatic cells (Díaz-Velasco et al. 2020; Estaras et al. 2020).

Among the assorted pathophysiological conditions induced by the intake of oxidized proteins and amino acids, the disturbance of the microbiota has been found in both in vitro (Arcanjo et al. 2019) and in vivo (Goethals et al. 2020) studies. The protective role of microbiota is gaining interest since luminal oxidative stress in humans can be counteracted by microbiota (Spyropoulos et al. 2011). In this regard, *Lactobacillus reuteri*, a natural colonizer of the gastrointestinal tract in humans and animals, has been used as a dietary supplement to enhance human gut health (Shornikova et al. 1997), and its oral administration reduces gastrointestinal disorders and infections and contributes to a balanced colonic microbiota (Shornikova et al. 1997). *L. reuteri* has been reported to protect against oxidative stress and inhibits the accretion of oxidation products in the lumen, according to the mechanisms related to its probiotic effects (Amaretti et al. 2013). While the benefits of *L. reuteri* against oxidative stress are documented (Petrella 2016), the molecular mechanisms implicated in the responses of this probiotic bacterium under specific pro-oxidant conditions are not well understood.

According to some previous reports, the expression of the *uspA* and *dhaT* genes in *L. reuteri* is affected by the oxidative threat caused by reactive oxygen species (ROS) (Arcanjo et al. 2019). Usp proteins seem to be implicated in the defense against DNA-damaging agents while the *dhaT* gene encodes for a propane-1,3-diol (1,3-PDO) oxidoreductase, which is involved in the protection of *L. reuteri* against oxidative stress (Arcanjo et al. 2019). In a preceding study, we investigated the molecular responses of this bacterium to a major lipid oxidation product, malondialdehyde (MDA), at concentrations between 5 and 100 μM (Padilla et al. 2021). Yet, the underlying mechanisms of the potential impact of oxidized amino acids on probiotic bacteria are unknown.

The aim of this study was to understand the molecular mechanisms activated in *L. reuteri* in response to the potential harmful effects of AAA. To fulfil this objective, the redox status (ROS generation and lipid and protein oxidation markers), and the expression of the *uspA* and *dhaT* genes in *L. reuteri* challenged by increasing concentrations of AAA, was investigated.

## Materials and methods

### Chemicals and raw material

Chemicals and reagents used in this study were of American Chemical Society analytical grade and purchased from Sigma Chemicals (Sigma–Aldrich, Germany), Scharlab S.L. (Spain), Pronadisa (Conda Laboratory, Spain), Applied Biosystems (USA), Epicentre (USA), and Acros Organics (Spain). *L. reuteri* PL503 was isolated from pig faeces and then identified using 16S rRNA gene sequencing by Ruiz-Moyano et al. (2008).

### Experimental setting

*L. reuteri* PL503 was stored at − 80 °C in Man Rogosa and Sharpe (MRS) broth with 20% (v/v) glycerol. To prepare the working cultures, *L. reuteri* PL503 was cultivated twice at 37 °C for 24 h in MRS broth supplemented with 0.5% acetic acid 10% (v/v). A volume of 100 µL of such culture of *L. reuteri* PL503 was inoculated in tubes of 5 mL of MRS broth containing different concentrations of free AAA. In particular, four groups were considered based on the added concentration of free AAA: Control (*L. reuteri*), 1 mM (*L. reuteri* + 1 mM AAA), 5 mM (*L. reuteri* + 5 mM AAA), and 10 mM (*L. reuteri* + 10 mM AAA). They were incubated at 37 °C for up to 24 h in microaerophilic conditions to simulate physiological conditions in the colon. The concentrations of free AAA are those expected to be found in the colon after gastrointestinal digestion of a severely processed muscle food (Utrera and Estévez 2012; Utrera et al. 2012; Goethals et al. 2020). For each treatment, three replicates were performed. During the incubation period, samples were taken at 12, 16, 20, and 24 h. For counting viable cells, 100 µL of *L. reuteri* PL503 of each treatment and sampling time were inoculated on MRS agar at the same sampling time and conditions as the experimental tubes. For protein analyses, to avoid possible contamination from the culture medium, two washes with phosphate buffered saline solution (PBS, pH 7.4) were made.

### Gene expression studies

#### RNA extraction

The RNA extraction of each experimental group and incubation time was performed using the MasterPure™ RNA purification kit (Epicentre), which includes DNase treatment. The obtained RNA was eluted in 35 μL TE buffer and kept at − 80 °C until further use. RNA quantity (ng/µL) and quality (A_260_/A_280_ ratio) were spectrophotometrically determined using the Nanodrop 2000 (Thermo Scientific, USA).

#### Reverse transcription reaction

The cDNA was synthesized using about 500 ng of total RNA, according to the PrimeScript™ RT Reagent kit (Takara Bio Inc., Japan). The cDNA was stored at − 20 °C until being used for the PCR reactions.

#### Real-time PCR analysis of gene expression

The *uspA* and *dhaT* genes were selected for relative expression studies using real-time PCR (qPCR), being used the 16S gene as reference gene. The amplification was performed in MicroAmp optical 96-well plates sealed with optical adhesive covers (Applied Biosystems) on a ViiA™ 7 Real-Time System (Applied Biosystems) using the SYBR Green technology. Each well contained 2.5 µL of cDNA, 6.25 µL of SYBR^®^ Premix Ex Taq™ (Takara Bio Inc.), 0.625 µL of ROX™ Reference Dye (Takara Bio Inc.), and 300 nM of each primer pair (Table 1). The qPCR program consisted of an initial denaturation step at 95 °C for 10 min; 40 cycles at a denaturation temperature of 95 °C for 15 s and annealing/extension temperatures of 55 °C and 60 °C for the 16S and target genes, respectively, during 30 s. After the final qPCR cycle, a melting curve was included by heating the product from 60 to 99 °C and continuous measurement of the fluorescence was performed to verify the qPCR products. All samples were analyzed in triplicate, including control samples consisting of adding sterile ultrapure water instead of cDNA. The expression ratio was calculated using the 2^−ΔΔC^_T_ method reported by Livak and Schmittgen (2001). The calibrator sample corresponded to the value of the expression of the experimental group Control at each sampling time.

**Table 1:** Sequences of primers used for reverse transcription real-time PCR assays to conduct gene expression analyses

Primers	Gene	Nucleotide sequence (5′-3′)	Annealing temperature	References
uspALr-F1	*uspA*	CTTGGGTAGCGTTCACCATT	60 °C	Arcanjo et al. (2019)
uspALr-R1		TGAAAAAGCGGTTGACACTG	60 °C	Arcanjo et al. (2019)
LS67	*dhaT*	TGACTGGATCCTAATTTGGTCCTGGTGTTATTGC	60 °C	Schaefer et al. (2010)
LS68		TGACTGAATTCTTCCGGATCTTAGGGTTAGG	60 °C	Schaefer et al. (2010)
Lr16S_F	16S rRNA	CCGCTTAAACTCTGTTGTTG	55 °C	Arcanjo et al. (2019)
Lr16S_R		CGTGACTTTCTGGTTGGATA	55 °C	Arcanjo et al. (2019)

### Analytical procedures

#### Analysis of ROS by flow-cytometry

Flow cytometry detection of ROS (e.g., hydroxyl and superoxide radicals) in *L. reuteri* PL503 was performed as determined using protocols described by Díaz-Velasco et al. (2020) with some minor modifications. In brief, samples of *L. reuteri* PL503 (1 × 10^6^ ufc/mL) of each experimental group and incubation time, were extended in 1 mL of PBS, and stained with CellRox Deep Red (5 μM; ThermoFisher, USA) (excitation and emission wavelengths, 644 and 645 nm, respectively) for detecting the bacterium producing ROS, and Hoechst 33,342 (0.5 μM; Sigma–Aldrich) (excitation and emission wavelengths, 345 nm and 488 nm, respectively) to identify the bacterium and remove debris from the analysis. After thorough mixing, the cell suspension was incubated at room temperature for 25 min in the dark, washed in PBS and immediately run on the flow cytometer. The analyses were conducted using a Cytoflex^®^ flow cytometer (Beckman Coulter, USA) equipped with violet, blue, and red lasers. The instrument was daily calibrated using specific calibration beads provided by the manufacturer. A compensation overlap was performed before each experiment; however, due to emission and excitation characteristics of the combination of the used probes, spectral overlap was negligible. Files were exported as FCS files and analyzed using FlowJoV 10.5.3 Software for Mac OS (Ashland, USA). Unstained, single-stained, and Fluorescence Minus One (FMO) controls were used to determine compensations and positive and negative events, as well as to set regions of interest.

#### Synthesis of allysine standard compound

N-Acetyl-L-AAS (allysine) was synthesized from Nα-acetyl-L-lysine using lysyl oxidase activity from egg shell membrane following the procedure described by Akagawa et al. (2002). Briefly, 10 mM Nα-acetyl-L-lysine was incubated at constant stirring with 5 g of egg shell membrane in 50 mL of 20 mM sodium phosphate buffer, pH 9.0 at 37 °C for 24 h. The egg shell membrane was then removed by centrifugation and the pH of the solution adjusted to 6.0 using 1 M HCl. The resulting aldehydes were reductively aminated with 3 mM 4-aminobenzoic acid (ABA) in the presence of 4.5 mM sodium cyanoborohydride (NaBH_3_CN) at 37 °C for 2 h with stirring. ABA derivatives were then hydrolyzed by 50 mL of 12 M HCl at 110 °C for 10 h. The hydrolysates were evaporated at 40 °C *in vacuo* to dryness. The resulting allysine–ABA was purified using silica gel column chromatography and ethyl acetate/acetic acid/water (20:2:1, v/v/v) as elution solvent. The purity of the resulting solution (70%) and authenticity of the standard compounds obtained following the aforementioned procedures were checked using MS and ^1^H NMR (Estévez et al. 2009).

#### Quantification of allysine

Allysine was quantified in bacterial protein as a marker of oxidation-induced post-translational modification, according to the method described by Utrera et al. (2011). Five hundred μL of each experimental group and incubation time of culture were dispensed in 2 mL microtubes and treated with cold 10% (v/v) trichloroacetic acid (TCA) solution. Each microtube was vortexed and then subjected to centrifugation at 600 × *g* for 5 min at 4 °C. The supernatants were removed, and the pellets were incubated with the following freshly prepared solutions: 0.5 mL 250 mM 2-(N-morpholino) ethanesulfonic acid (MES) buffer pH 6.0 containing 1 mM diethylenetriaminepentaacetic acid (DTPA), 0.5 mL 50 mM ABA in 250 mM MES buffer pH 6.0 and 0.25 mL 100 mM NaBH_3_CN in 250 mM MES buffer pH 6.0. After vortexing, the tubes were incubated in a water bath at 37 °C for 90 min. The samples were stirred every 15 min. The samples were then treated with a cold 50% TCA (v/v) solution and centrifuged at 1200 × *g* for 10 min. The pellets were washed twice with 10% TCA and diethyl ether-ethanol (1:1). Finally, the pellet was treated with 6 M HCl and kept in an oven at 110 °C for 18 h until completion of hydrolysis. The hydrolysates were dried *in vacuo* in a centrifugal evaporator. The generated residue was reconstituted with 200 µL of milliQ water and then filtered through hydrophilic polypropylene GH Polypro (GHP) syringe filters (0.45 μm pore size, Pall Corporation, USA) for HPLC analysis.

A Shimadzu ‘Prominence’ HPLC apparatus (Shimadzu Corporation, Japan), equipped with a quaternary solvent delivery system (LC-20AD), a DGU-20AS on-line degasser, a SIL-20A auto-sampler, a RF-10A XL fluorescence detector (FLD), and a CBM-20A system controller, was used. An aliquot (1 μL) from the reconstituted protein hydrolysates was injected and analyzed in the HPLC-FLD equipment. Allysine–ABA was eluted in a Cosmosil 5C_18_-AR-II RP-HPLC column (5 µm, 150 × 4.6 mm) equipped with a guard column (10 × 4.6 mm) packed with the same material (Phenomenex, PA, USA). The flow rate was kept at 1 mL/min and the temperature of the column was maintained constant at 30 °C. The eluate was monitored with excitation and emission wavelengths set at 283 and 350 nm, respectively. Standards (0.1 μL) were run and analyzed under the same conditions. Identification of both derivatized semialdehydes in the chromatograms was carried out by comparing their retention times with those from the standard compounds. The peak corresponding to allysine–ABA was manually integrated from the FLD chromatograms and the resulting areas plotted against an ABA standard curve with known concentrations that ranged from 0.1 to 0.5 mM (Utrera et al. 2011). Results were expressed as nmol of allysine per mg of protein.

#### Analysis of Schiff bases

The formation of Schiff bases in bacterial protein was assessed in each experimental group and incubation time by fluorescence spectroscopy. Prior to the analysis, reaction mixtures were diluted (1:20) with 8 M urea in 100 mM sodium phosphate buffer, pH 7. Diluted samples were dispensed in spectrofluorometric cuvettes and excited at 350 nm using a LS-55 Perkin–Elmer fluorescence spectrometer (PerkinElmer, UK). The fluorescence emitted by Schiff bases was recorded at 450 nm. The excitation and emission slit widths were set at 10 nm and the speed of data collection while scanning was of 500 nm per min. The height of the peaks corresponding to Schiff bases spectra was recorded. After taking into consideration the applied dilutions, the results were expressed as fluorescence units.

#### Analysis of protein thiols

Thiols from sulfur-containing amino acids in bacterial proteins were quantified in accordance to the method reported by Rysman et al. (2014). A volume of 250 µL of each *L. reuteri* PL503 experimental group and incubation time, was washed twice with PBS and ethanol:ethyl acetate (1:1) to avoid possible contamination with thiols from the medium. Upon centrifugation (600 × *g*/5 min), the pellet was resuspended in 250 µL of guanidine hydrochloride, treated with 250 µL of 4,4′-dipyridyldisulphide (DPS) in 12 mM HCl and dispensed in a spectrophotometric cuvette. Absorbance was measured at 324 nm against a blank sample in which DPS was replaced with an equivalent volume of guanidine hydrochloride. Quantification was made by preparing a standard curve with cysteine. The results were expressed as µmol of free thiol groups per mg of protein.

#### Analysis of thiobarbituric-reactive substances

The quantification of MDA and other thiobarbituric-reactive substances (TBARS) in all experimental groups and incubation times, was made in accordance to the method described by Ganhao et al. (2011). An aliquot of 200 µL of *L. reuteri* PL503 experimental group was treated with 500 µL of thiobarbituric acid (0.02 M) and 500 µL of TCA (10%) and incubated during 20 min at 90 °C. After cooling, a 5 min centrifugation at 600 × *g* was made and the supernatant was measured at 532 nm. Quantification was made by preparing a standard curve with tetraethoxypropane (TEP). The results are expressed as mg TBARS per L of sample.

### Statistical analysis

True replicates (*n* = 3) were subjected to duplicate analyses and data were collected and subjected to statistical analysis. Earlier, the data were analyzed for normality (Shapiro–Wilk test) and homoscedasticity (Bartlett test). The effects of AAA concentration and incubation times were studied using analyses of variance (ANOVA) (SPSS v. 15.5). The effect of AAA on the gene expression (ΔΔC_T_ values) was analyzed using the paired Students’ *t* test (SPSS v. 15.5). The statistical significance was set at *p* ≤ 0.05.

## Results

### Relative expression of the uspA gene

**Fig. 1:**
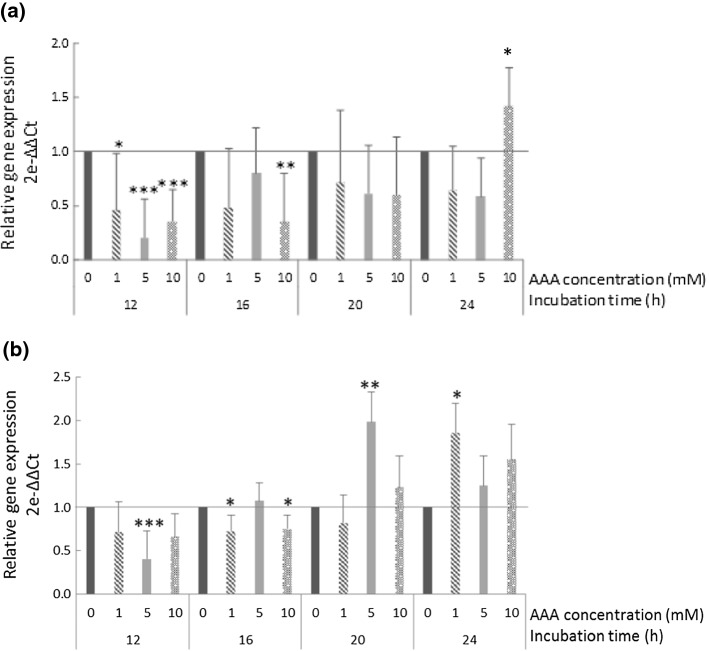
Relative expression (2-^ΔΔC^_T_) of the *uspA* (**a**) and *dhaT* (**b**) genes in *Lactobacillus reuteri* PL503 grown in the presence of increasing concentrations (0, 1, 5, and 10 mM) of α-aminoadipic acid (AAA) for up to 24 h. Black line at 2-^ΔΔC^_T_ = 1 denotes standardized expression rate for CONTROL group (0 mM) at each sampling time (calibrator). 2-^ΔΔC^_T_ < 1 denotes suppression of the expression of the target gene; 2-^ΔΔC^_T_ > 1 denotes activation of the expression of the target gene. Asterisks on top of bars denote significant differences between such treatment and the control within a particular sampling time (*p ≤ 0.05; **p ≤ 0.01; ***p ≤ 0.001)

The relative expression of the *L. reuteri* PL503 *uspA* gene during the incubation assay in the presence of different concentrations of AAA is shown in Fig. 1a. A significant downregulation of the *uspA* gene was particularly observed at 12 h of incubation in the presence of 5 and 10 mM of AAA (0.22- and 0.40-fold decrease, respectively) as well as at 16 h sampling with 10 mM (0.38-fold decrease). An upregulation of the gene (1.43-fold increase) was observed at 24 h when the bacterium was exposed to the highest AAA concentration.

### Relative expression of the *dhaT* gene

The relative expression of the *L. reuteri* PL503 *dhaT* gene during the incubation assay in the presence of different concentrations of AAA is shown in Fig. 1b. A significant downregulation of the gene was found at 12 h in the presence of 5 mM (0.42-fold decrease) and at 16 h in the presence of both 1 mM and 10 mM of AAA (0.75- and 0.81-fold decreases, respectively). Nonetheless, in the two final sampling times, an upregulation of the relative transcription of the *dhaT* gene was observed. In particular, significant changes were found in the presence of 5 mM of AAA at 20 h (1.98-fold increase) and 1 mM of AAA at 24 h (1.83-fold increase).

### ROS generation by flow-cytometry analyses

**Fig. 2:**
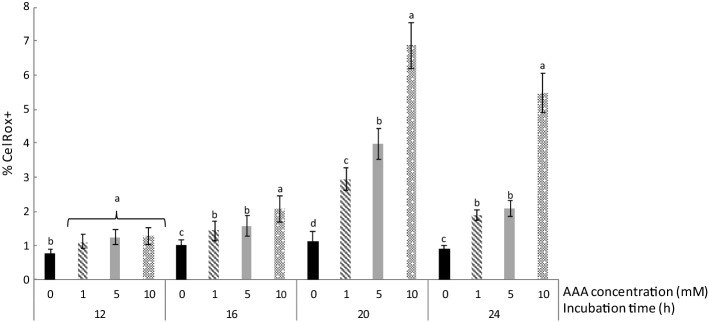
Percentage of *Lactobacillus reuteri* PL503 suffering from oxidative stress (positive to Cell Rox dye) when grown in the presence of increasing concentrations (0, 1, 5 and 10 mM) of α-aminoadipic acid (AAA) for up to 24 h. Different letters on top of bars denote significant differences (*p* ≤ 0.05) between AAA concentrations within the same sampling time

The incubation of *L. reuteri* PL503 in the presence of AAA led to an increased production of ROS as shown in Fig. 2. The analysis of the samples with flow-cytometry showed a clear dose effect. At increasing concentrations of AAA, the percentage of bacterium suffering oxidative stress at 24 h rise from 0.8% in control group to 1.8%, 2.1%, and 5.3% in bacteria exposed to 1, 5 and 10 mM AAA, respectively. Specially, at the two final sampling times, the differences between groups were found to be higher than in the previous ones.

### Analysis of thiobarbituric-reactive substances

**Fig. 3:**
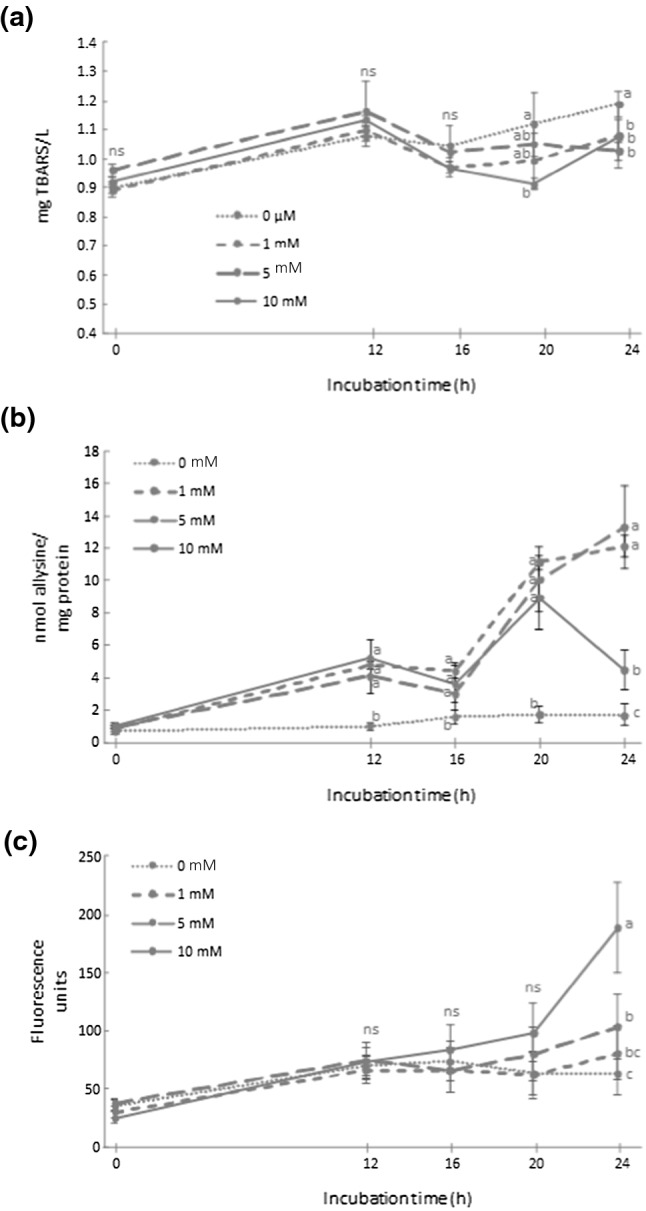
Concentration of thiobarbituric-reactive substances (TBARS) (**a**), allysine (**b**) and Schiff bases (**c**) (means ± standard deviation) in *Lactobacillus reuteri* PL503 grown in MRS broth in the presence of increasing concentrations (0, 1, 5 and 10 mM) of α-aminoadipic acid (AAA) during an incubation period for up to 24 h. Different letters at the same sampling time denote significant differences between AAA concentrations (*p* ≤ 0.05)

In Fig. 3a, the TBARS concentration in *L. reuteri* PL503 during the assay is shown. In the presence of AAA, significant changes occur at 20 h with 10 mM of AAA lowering TBARS content compared to control samples (0.89 mg TBARS/L vs. 1.10 mg TBARS/L). At 24 h, the concentration of TBARS in control samples (1.17 mg TBARS/L) was significantly higher than in the bacterium challenged with AAA (ranging from 1.05 to 1.10 mg TBARS/L).

### Quantification of allysine

The changes of the concentration of allysine in *L. reuteri* PL503 during the incubation period is shown in Fig. 3b. Compared to control, the exposure to AAA caused a significant increase in the concentration of allysine in proteins from *L. reuteri* PL503 for 20 h. At that sampling point, the concentration of allysine in control samples (1.8 nmol/mg protein) was significantly lower than in those treated with 1, 5, and 10 mM AAA (11.7, 10.4, and 8.8 nmol/mg protein, respectively). At 24 h sampling, the behavior varied between groups. In *L. reuteri* challenged with 1 and 5 mM of AAA, the increase of allysine was constant during the complete assay reaching the highest concentration at 24 h (12.0 and 13.5 nmol/mg protein, respectively). On the other hand, when the bacterium was exposed to the highest AAA concentration (10 mM) allysine peaked at 20 h, after which a decrease was observed at the end of the incubation period (4.2 nmol/mg protein).

### Analysis of Schiff bases

In the present study, the formation of Schiff bases is shown in Fig. 3c and a clear dose effect of AAA was observed. No significant differences were found between AAA concentrations during the first three sampling times. Nevertheless, at the final sampling time (24 h) an increase was observed when the bacterium was exposed to the highest concentration (10 mM), which is coincident with carbonyls depletion found in the same group of samples at the end of the assay. At 24 h, the relative concentration of Schiff bases in *L. reuteri* followed the increasing order: control group (52 fluorescent units) and bacterium exposed to 1, 5 and 10 mM AAA (80, 98, and 185 fluorescent units).

### Analysis of protein thiols

**Fig. 4:**
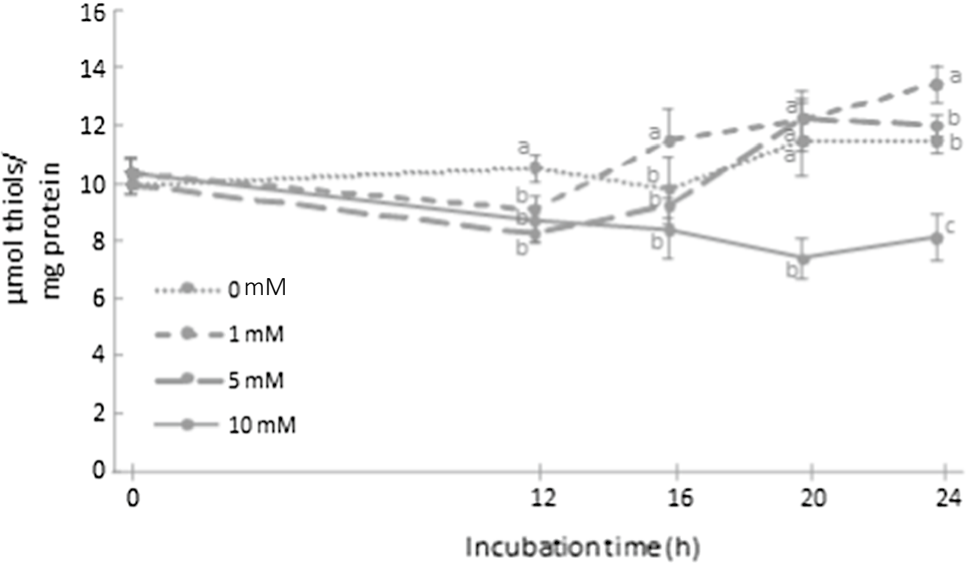
Concentration of free thiols (means ± standard deviation) in *Lactobacillus reuteri* PL503 grown in MRS broth with increasing concentrations (0, 1, 5, and 10 mM) of α-aminoadipic acid (AAA) during an incubation period for up to 24 h. Different letters at the same sampling time denote significant differences between AAA concentrations (*p* ≤ 0.05)

The concentration of free thiols in proteins from *L. reuteri* PL503 during the incubation assay is shown in Fig. 4. Significant differences were observed in the first 12 h between the control group and the bacterium challenged with increasing AAA concentrations. From 16 h sampling time onwards, a significant increase of free thiols in samples exposed to 1 and 5 mM of AAA was detected, peaking at 24 h concentrations of 13.3 μmol/mg protein and 11.8 μmol/mg protein, respectively. Conversely, the concentration of thiols in bacteria exposed to the highest AAA concentration (10 mM) significantly decreased from the first sampling (10 μmol/mg protein) until the end of the assay (7.9 μmol/mg protein).

## Discussion

### Regulation of the *uspA* and *dhaT* genes by *L. reuteri* in response to AAA

*L. reuteri* counts remained stable with the increasing applied doses of AAA (1 mM, 5 mM, and 10 mM) during the entire experimental assay (37 °C/24 h), so the survival was not jeopardized (data not shown). Yet, the challenge with this oxidized amino acid led to impairments of the bacterium’s physiology. This finding reflects the ability of *L. reuteri* to activate mechanisms to neutralize the potential harmful effects of the sub-lethal concentrations of the added oxidized amino acid. In the present study, these mechanisms were firstly assessed by the analysis of the relative expression of stress-related genes.

The universal stress protein A (UspA) superfamily includes an ancient and conserved group of proteins found in assorted microorganisms, insects, and plants. The precise roles of Usp proteins in biological systems remain unclear; yet, they seem to be involved in the defense against DNA-damaging agents and respiratory uncouplers (Kvint et al. 2003). Due to the defined function of the gene *uspA*, an upregulation was expected, which was only observed at 24 h and in the presence of the highest AAA concentration (Fig. 1a). Yet, the downregulation observed at earlier samplings and lower concentrations is consistent with data reported by Oberg et al. (2015), who found a significant downregulation of the *uspA* gene expression in *Bifidobacterium longum* exposed to a hydroxyl-radical generating system. Similar results were reported by Arcanjo et al. (2019) working on the same bacterium and strain from the present study. In that study, exposing *L. reuteri* to 0.5 mM of hydrogen peroxide led to a significant decrease of the *uspA* gene expression. It is worth noting that both aforementioned studies found the occurrence of oxidative stress and molecular damage in the exposed bacteria. The fact that AAA exposure led to a similar effect on *L. reuteri* indicates that this oxidized amino acid is identified by the bacterium as a chemical threat. In fact, two recent studies agree in describing noxious effects of food-compatible AAA concentrations (200 μM) on human intestinal (Díaz-Velasco et al. 2020) and human acinar pancreatic cells (Estaras et al. 2020). According to these authors, the harmful effect of AAA involved the induction of pro-oxidative conditions within cells. Probiotic bacteria like *L. reuteri* may also be susceptible to this chemical species and*,* according to these results, the downregulation of *the uspA* gene seems to be related to a cellular signal of a pro-oxidative threat that both, the radical generating systems (i.e., hydrogen peroxide) and oxidized amino acids such as AAA, may be able to induce.

It is worth clarifying that the higher AAA concentrations tested in the present study (1–10 mM) are plausibly compatible with a physiological situation as explained as follows. While AAA concentration in foods has been found to reach up to 200 μM, it is also known that dietary proteins are further oxidized during digestion, increasing significantly the final concentration of oxidized amino acids in the gut. For instance, in a study by Van-Hecke et al. (2019), the concentration of protein oxidation products increased between 2 and fivefold times in assorted foods after simulated gastrointestinal digestion. The same authors found in a more recent study (Goethals et al. 2020) sixfold times higher concentrations of protein oxidation products in pork digests than in the original (undigested) pork product.

The *dhaT* gene encodes the enzyme 1,3-PDO oxidoreductase which is known to play a relevant role in stressful situations involving energetic demand. This enzyme enables the main carbohydrate fermentation pathway (6-phosphogluconate/phosphoketolase; 6-PG/PK) through the production of NAD^+^ (required for glucose fermentation) from NADH in the conversion of 3-hydroxypropionaldehyde (3-HPA) (its substrate) into 1,3-PDO under anaerobic conditions. Additionally, 3-HPA, also known as reuterin, is excreted by *L. reuteri* strains under stressful situations (Schaefer et al. 2010). The overexpression of this gene observed in bacteria exposed to 5 and 1 mM AAA for 20 and 24 h, respectively, could respond to an attempt of the bacteria to protect against the oxidative threat caused by this oxidized amino acid. The elemental mechanisms by which *L. reuteri* may seek to protect against AAA-induced biological damage through the activation of the 3-HPA pathway should be subjected to scrutiny. As previously reported by Talarico et al. (1988), the 3-HPA pathway requires glycerol, commonly added as growth promoter in *Lactobacillus* cultures. In the present study, *L. reuteri* had no access to such precursor, and, therefore, the 3-HPA pathway is unlikely to have occurred. Considering the absence of glycerol, it seems reasonable to consider that 1,3-PDO may have other substrates and that its cellular activity may be related to protection against a potential pro-oxidative threat. To similar conclusions came Arcanjo et al. (2019) who found an increased expression of the *dhaT* gene in *L. reuteri* challenged with hydrogen peroxide in simulated colonic conditions where glycerol was, again, absent. The authors hypothesized whether the NAD + -dependent activity of the 1,3-PDO may be able to detoxify hydrogen peroxide in the presence of NADH. Since no hydrogen peroxide was included in the present assay, the implication of 1,3-PDO in balancing the redox state of the cell seems to be a pertinent defense mechanism against pro-oxidative threats. It is, still unknown how AAA may impair the redox status of *L reuteri* but it is proven that AAA exposure to human eukaryotic cells cause oxidative stress via mitochondrial disturbance and ROS generation (Díaz-Velasco et al. 2020; Estaras et al. 2020).

It is worth noting that the effect of AAA exposure on the expression of the *dhaT* gene at early stages of the assay (12 and 16 h) was opposite to that observed at advanced stages. As discussed in due course, the activation of the gene at advanced stages of oxidative stress and oxidative damage could have triggered defense mechanisms, in which the *dhaT* gene may be implicated. At early stages, the underexpression of this gene could respond to indefinite initial responses of the bacteria to the AAA exposure, in which the protein encoded by this gene was not found as essential. In line with this downregulation, a recent study by Díaz-Velasco et al. (unpublished data) observed that AAA exposure to CACO-2 cells led to an overall downregulation of gene expression due to the impairment of protein kinase A and C (PKA and PKC, respectively) signaling pathways. Yet, the mechanisms implicated in the downregulation of *dhaT* gene at early stages of exposure to AAA in this bacterium remain indefinite and require further elucidation.

### ROS generation in *L. reuteri* by AAA

The increased production of ROS in *L. reuteri* by the presence of AAA has no precedent in literature (Fig. 2). It is, however, consistent with results reported by Díaz-Velasco et al. (2020) in CACO-2 cells and Estaras et al. (2020) in pancreatic cells when the exposure to AAA led to impairment of the oxidative status of the cell, ROS generation, apoptosis, and necrosis. In addition, it is in accordance to Da Silva et al. (2017) who studied the effect of AAA on brain function of adolescent rats, and showed an induction of ROS generation and alteration of the cellular redox status via mitochondrial impairment. While the percentage of *CelRox* positive bacteria was found to be relatively low, previous studies using hydrogen peroxide and malondialdehyde (MDA) as inductors of oxidative stress in *L. reuteri* reported similar percentages (Arcanjo et al. 2019; Padilla et al. 2021). The oxidative damage caused in bacterial lipids and proteins, explained in due course, denote severe oxidative stress. The precise mechanisms by which AAA is able to induce ROS generation in *L. reuteri* are indefinite. It is worth noting that such mechanisms differ from those reported by the aforementioned authors since the bacterium lacks mitochondria. Interestingly, *Lactobacillus spp.* have also been found to be able to produce hydrogen peroxide and other ROS via implication of NAD(P)H oxidoreductases (Hertzberger et al. 2014) which provides a plausible and coherent connection between AAA exposure, *dhaT* overexpression and ROS generation. The molecular mechanisms underlying the interconnection between all these elements need to be precisely described.

### Oxidative damage to *L. reuteri* by AAA

In the present work, the oxidative damage to bacterium caused by AAA-induced oxidative stress was assessed by means of TBARS (lipid oxidation) and allysine (protein oxidation). The basal TBARS concentration in control cultures, (~ 1 mg/L) may correspond to the occurrence of lipid peroxidation in the bacterium under physiological conditions and did not change significantly during the assay within groups (Fig. 3a). AAA did not significantly affect the extent of lipid oxidation in *L. reuteri.*

On the other hand, AAA exposure had a significant impact on the oxidative damage to bacterial proteins. A relatively low but significant increase in allysine, the main protein carbonyl in biological systems (Stadtman and Levine 2000; Estévez and Luna 2016), was observed in the control group of *L. reuteri* (Fig. 3b). The present results show that allysine, formed in bacteria, as in eukaryotes, remarkably contributes to protein carbonylation and may be used as a reliable indicator of oxidative stress. The results obtained are in accordance with Ezraty et al. (2017) who proposed that protein carbonylation could be a reflection of bacterial senescence as oxidized proteins accumulate in non-proliferating bacteria. Allysine is typically formed in proteins because of the attack of ROS to lysine residues. This is plausibly the mechanism taking place in the present assay as the significant production of ROS in *L. reuteri* exposed to AAA exposure could have caused the oxidation of lysine residues and hence, the accretion of allysine. Once formed, allysine may also react with amino groups from neighboring amino acids (e.g., lysine) to form an azomethine structure, also known as Schiff bases (Estévez 2011). The dramatic drop of allysine concentration during the last 4 h of the assay in the bacterium exposed to the highest concentration of AAA (10 mM) is consistent with the sudden increase of Schiff bases in that period of time (Fig. 3c). These results suggest that such fluorescent structures were, at least, partially formed in bacteria exposed to 10 mM as a result of allysine addition to other protein amines. The formation of Schiff bases in bacteria exposed to intermediate AAA doses (1 and 5 mM), was not so intense to reflect a decline of the reactant (allysine). Both, carbonylation and formation of non-reducible protein cross-links (i.e., Schiff bases), are irreversible protein modifications with negative biological consequences (Davies 2005; Ezraty et al. 2017; Estévez and Xiong 2019). Carbonylated proteins can be dysfunctional and may be labeled to removal due to its accumulation causes impaired homeostasis that leads to chronic dysfunction and apoptosis (Shacter 2003). However, carbonylated proteins can also act as signaling molecules, which may activate specific pathways, to preserve homeostasis control senescence (Shacter 2003).

Both situations could be applied to the present experiment. The increase in carbonyls above 10 nmol/mg proteins in the bacterium challenged with 5 and 1 mM of AAA was coincident with the activation of the *dhaT* gene at sampling times of 20 h and 24 h, respectively, and plausibly, the corresponding synthesis of the NADH-dependent oxidoreductase decoded by this gene. Given the proposed role of this enzyme in detoxifying pro-oxidant species (Arcanjo et al. 2019), a relatively mild pro-oxidative threat, exhibited in a significant accretion of protein carbonyls, could have led to the activation of an antioxidant response mediated, among others, by the activation of the *dhaT* gene. On the other hand, a severe oxidative damage caused by a more intense pro-oxidative environment, such as that observed in *L. reuteri* challenged with the highest concentration of AAA (10 mM) led to a sudden formation of advanced oxidation products (Schiff bases) and no *dhaT* gene-mediated response against the oxidative insult. These mechanisms were not present in the bacterium incubated with the lowest doses of AAA (1 and 5 mM). Previous considerations made by Ezraty et al. (2017) and Arcanjo et al. (2019) support the hypothesis that the *dhaT* gene could have been activated by pro-oxidant species and/or the effect of the former on protein carbonylation.

The evolution of protein thiols during the assay (Fig. 4) provides additional strength to the aforementioned hypotheses. The oxidation of sulfur-containing amino acids, such as cysteine (Cys) and methionine (Met), is a typical feature in biological systems attacked by ROS (Estévez et al. 2020). While the oxidation of thiols in proteins may lead to dysfunction, irrelevant sulfur-containing amino acids are known to act as antioxidants offering a sacrificial loss to ROS and protecting other amino acids with relevant significance, such as lysine (Davies, 2005; Estévez et al. 2020). This dual role of thiols was examined in the present experiment. Taking into account that these moieties can act as redox-active compounds and elements of antioxidant protection in biological systems, the coincidence of thiol accretion with the increase of carbonylation in those samples may respond to a strategy to keep a balanced redox status in cells in danger. The incubation of *L. reuteri* with AAA caused an increase of thiol concentration since 12 h incubation onwards. The pro-oxidant changes induced by AAA, including the formation of protein carbonyls, possibly triggered the accumulation of thiol groups by the novo synthesis of sulfur-containing proteins/peptides with the purpose of protecting the bacterium against this pro-oxidant threat. Thiol accumulation is considered as an endogenous mechanism of antioxidant defense owing to the recognized redox-active properties (Davies 2005). These moieties have been typically regarded as elements of antioxidant protection in eukaryotes and in lactic acid bacteria (Schaefer et al. 2010; Xiao et al. 2011). However, the molecular mechanism backing the synthesis of thiol-containing species remain unclear and needs further clarification. It is worth noting that such thiol accretion did not take place in cultures treated with 10 mM of AAA, confirming the lack of genetic (*dhaT* mediated) and antioxidant response in these bacteria. The irreversible loss of thiols in this group of bacteria may respond to the consumption of these moieties in the severe pro-oxidative environment caused by 10 mM of AAA.

## Conclusions

The present results show, for the first time, that a food-occurring oxidized amino acid, the AAA, is able to disturb the redox balance of the probiotic bacterium *L. reuteri* by inducing the formation of ROS and causes protein oxidative damage. This bacterium seems to be able to activate both genetic and molecular mechanisms to struggle with the oxidative threat. The *dhaT* gene is proposed to play a role by encoding a NAD + -dependent oxidoreductase that may contribute to detoxify oxidizing species. The specific effects exerted by the highest AAA concentrations are more unlikely to be occur in physiological conditions while the exact amount of free AAA in food digests is yet to be defined. Finally, the present results and their consequences for the microbiota and the impact on the host may be further studied in upcoming in vivo studies.

## Abbreviations

1,3-PDO: Propane-1,3-diol
3-HPA: 3-Hydroxypropionaldehyde
AAA: α-Aminoadipic acid
AAS: α-Aminoadipic semialdehyde
ANOVA: Analyses of variance
DNA: Deoxyribonucleic acid
ABA: Aminobenzoic acid
DPS: 4,4′-Dipyridyldisulphide
DTPA: Diethylenetriaminepentaacetic acid
FLD: Fluorescence detector
FMO: Fluorescence minus one
HPLC: High-performance liquid chromatography
MDA: Malondialdehyde
MES: 2-(N-morpholino)ethanesulfonic acid
MRS: Man Rogosa and Sharpe
NADH: Nicotinamide adenine dinucleotide
PBS: Phosphate buffered saline solution
PCR: Polymerase chain reaction
ROS: Reactive oxygen species
RNA: Ribonucleic acid
TBARS: Thiobarbituric-reactive substances
TCA: Trichloroacetic acid
TEP: Tetraethoxypropane
UspA: Universal stress protein A
